# 

**Published:** 1888-03

**Authors:** 


### BOOK NOTICES.

### NEW EXCHANGES ETC.

LACTOPEPTINE MEDICAL ANNUAL.—This is an illustrated pamphlet of 64 pages, published by "The N. Y. Pharmacal Association ;" the manufacturers of the reliable digestive agent known as 'Lactopeptine,' which has justly won its way to great appreciation, as a standard specific for impaired digestion. We cheerfully recommend its use after a careful observance of its value to the medical profession.

THE JOURNAL OF HYGEIO THERAPY.—Monthly, Kokomo, Ind. $1, per annum. An admirable exponent of the anti-drug system of health and cure.

THE MONITOR.—Monthly. Henderson, N. C. $1 per annum. Abounds in well selected and original matter for home reading.

OUR LITTLE MEN AND WOMEN.—Monthly—Boston—D. Lathrop Co. $1 per annum. A most excellent and beautifully illustrated monthly for little folk ; immensely popular.

BABYLAND.—Is another monthly of the same order by the same company. 50 cts. per annum, for babies and nobody else. A great encouragement to this popular enterprise, with mother goosey rhymes and pictures, no baby should fail to subscribe for it.

CHILDREN'S FRIEND AND KINDERGARTEN.—Monthly. 33 East 22d. Street, N. Y. City—For youth, excellent in its way, $1.50 per annum.

### OLD FRIENDS.

THE CENTURY for February, made its appearance on our table, as innocent and well conditioned as if it had not given us the go by for January. Where everything in it is so good, it is unnecessary to particularise. The MARCH number contains the true story of "Col. Rose's Tunnel at Libby Prison" told by one of the 125 escaped Union Officers. This monthly is just the kind one cannot do without, after having once had a taste of it. The Century Co. N. Y. City.

THE GALVANO CAUTERY SOUND.—An instrument in aid of surgery, invented by Robert Newman M. D. of N. Y. City, is fully explained in a pamphlet issued by that gentleman, with testimonials as to its efficacy in numerous instances.

THE COSMOPOLITAN.—N. Y. Schlicht and Field Co.—$2 per annum. A new and interesting feature of this well stored monthly, is the colored plates delicately executed, which embellish its pages. The " Ballet in Paris," so illustrated leads the February number.

WOMAN.—N. Y. Monthly, $2.75 per annum. Is a most praiseworthy enterprise as one of its aims is to assist in making young women useful and self-supporting. Its leading article for February, is a very full description of " The Young Woman's Christian Association," of N.Y. City amply illustrated, wherein are taught many useful and artistic employments, including drawing, painting, type writing, and stenography.

THOUGHT OF THE TIMES—N. Y. City—Monthly, $1 per annum, "Devoted to Stirring Topics of To-day." We notice that the well-known writer and essayist S. H. Preston, late associate Editor of " Earnest Words," is in the lead of this new enterprise. From our knowledge of the ability, liberality of sentiment, and literary resources of this gentleman, we do not hesitate to predict for this new applicant for pnblic favor a career of great usefulness, as well as prosperity.

TABLE TALK—Monthly, Philadelphia—$1 per annum. Devoted to the needs of American Housewives, Cookery &c. None better. Mrs. S. T. Rover, President Pha. Cooking School, Editor.

THE ESOTERIC—This monthly as its name implies, is devoted to the unseen, but ever potent forces which surround and enter into our respective individualities. The leading article of the February number is a brief treatise upon "Aura," an element much commented upon, but scarcely ever comprehended. Published at 478 Shawmut Avenue, Boston—$1.50 per annum.

VICK'S ILLUSTRATED MONTHLY, and the HORTICULTURAL ART JOURNAL, both of Rochester, are as usual, invaluable to Fruit and Flower culturists. Both are beautifully illustrated.

